# Levodropropizine for treating cough in adult and children: a meta-analysis of published studies

**DOI:** 10.1186/s40248-015-0014-3

**Published:** 2015-05-31

**Authors:** Alessandro Zanasi, Luigi Lanata, Giovanni Fontana, Federico Saibene, Peter Dicpinigaitis, Francesco De Blasio

**Affiliations:** Pneumology Unit, University of Bologna, S. Orsola Malpighi Hospital, Bologna, Italy; University Hospital Careggi, Florence, Italy; Medical Department, Dompé SPA, Milan, Italy; Respiratory Medicine and Pulmonary Rehabilitation Section, Clinic Center, Private Hospital, Naples, Italy; Department of Medicine, Albert Einstein College of Medicine and Montefiore Medical Center, Bronx, NY USA

## Abstract

**Background:**

Cough is one of the most common symptoms for which patients seek medical attention from primary care physicians and lung specialists. About 40% of the population at any one time report cough.

Cough is associated with significantly impaired health-related quality of life.

Levodropropizine is an effective and very well tolerated peripheral antitussive drug. We want to compare it to central cough suppressants efficacy (opioids and non-opioids) that may be associated with side effects limiting their use.

**Methods:**

After a comprehensive literature search, a meta-analysis of 7 clinical studies of levodropropizine vs. control, including a total of 1,178 patients, was performed with the aim to evaluate the overall comparative efficacy of levodropropizine in the pediatric and adult population.

Three electronic databases and reference list were used to search for studies that assessed the efficacy of levodropropizine for treating cough in children and adults using as standardized efficacy parameters the cough frequency and severity, and number of night awakenings as outcome parameters.

**Results:**

The meta-analysis of all standardized efficacy parameters showed a highly statistically significant difference in the overall antitussive efficacy in favor of levodropropizine vs. control treatments (p = 0.0015).

The heterogeneity test for the efficacy outcome was not statistically significant (p = 0.0534).

Seven studies met out inclusion criteria. A meta-analysis of the eligible ones showed a statistically significant difference in the overall anti-tussive effect of levodropropizine versus control (p = 0.0015).

**Conclusions:**

This analysis indicates that levodropropizine is an effective antitussive drug in children and adults, with statistically significant better overall efficacy outcomes vs. central antitussive drugs (codeine, cloperastine, dextromethorphan) in terms of reducing cough intensity and frequency, and nocturnal awakenings. This result further reinforces the favorable benefit/risk profile of levodropropizine in the management of cough. The efficacy of levodropropizine in the treatment of cough in children and adults is higher than that of the common centrally-acting anti-tussive.

## Background

Cough is one of the most common symptoms for which patients seek medical attention from primary care physicians and lung specialists [1]. In epidemiologic studies, up to 40% of people at any one time report cough [2].

Clinically, the etiology of cough can be broadly classified into acute and chronic as deduced from the length of time it persists, acute cough lasting from 1 to 3 weeks and chronic cough lasting more than 8 weeks. The most frequent causes of acute cough are viral or bacterial upper respiratory tract infections (URTIs), while those of chronic cough are asthma, gastro-esophageal reflux disease (GERD), chronic rhinitis, chronic bronchitis, chronic obstructive pulmonary disease (COPD), ACE-inhibitors treatment [3].

Regardless of whether it is acute or chronic, cough is associated with significantly impaired health-related quality of life, as sleep disturbance, nausea, chest pain and lethargy frequently occur [2].

The general approach managing any cough begins with a search for the cause of cough and treatment of the underlying cause. However, to recognize the origin of cough is not always an easy task and, even when identified, cough is refractory to specific therapy in a significant number of patients [1]. Furthermore, empiric treatment with antitussive agents is often needed, in particular when associated with deterioration in the quality of life [1, 2].

The etiology of cough in children differs from that in adults: viral URTI, protracted bacterial bronchitis and asthma are frequently the cause of cough [2] in children. So, the empirical approach commonly used in adults is unsuitable for children. Clinical evaluation of cough in children should also include an assessment of environmental factors, particularly tobacco smoke, parental concerns and expectations [4].

Two types of antitussive drugs are mainly available for the management of cough: centrally acting (opioids and non-opioids) cough suppressants and peripheral antitussives. Codeine, dextromethorphan and cloperastine are among the most common central agents that inhibit cough primarily by their effect on the cough center. Levodropropizine is a non-opioid agent whose peripheral antitussive action may result from its modulation of sensory neuropeptide levels within the respiratory tract [5]. In particular, levodropropizine exerts its antitussive effect through an inhibitory action at the level of the airway sensory nerves and it has been shown to be able to inhibit *in vitro* the release of neuropeptides from C-fibers [6]. In addition, in anaesthetized cats, it markedly reduces the activation of C-fibres and abolishes the associated reflexes [7]. The activity of levodropropizine on airway sensory units other than the C-fibres has not been investigated.

Centrally acting cough suppressants, although largely used, may achieve antitussive activity at the expense of unpleasant or intolerable side effects in adults and serious adverse events in children: side effects like drowsiness, dependency, loss of awareness, insomnia and difficulty in breathing [2].

Furthermore, the efficacy of most antitussive drugs, particularly those for URTI, has been challenged recently; in fact, the American College of Chest Physicians (ACCP) advises against the use of antitussive drugs in URTI [2].

Thus, the aim of the present study was to make a meta-analysis of clinical studies of levodropropizine vs. control drugs to evaluate the overall comparative efficacy of levodropropizine in the pediatric and adult population.

## Methods

### Literature search and study selection

A comprehensive systematic literature search was carried out on the main scientific electronic databases (PubMed/MEDLINE, EMBASE, and Cochrane Library) from their inception throughout May 2014, to identify original clinical studies of levodropropizine for the treatment of cough in the pediatric and adult settings. We sought additional articles from reference lists of review articles.

The inclusion criteria used to select studies were established *a priori*. Only studies with a controlled design (vs. both active control and placebo), including pediatric and adult patients and assessing efficacy endpoints related to cough outcomes, were selected.

Out of all the studies identified by means of our systematic literature search a total of 7 published clinical studies conducted with levodropropizine in adults or children met the eligibility criteria and were selected for our meta-analysis.

These studies included a total population of 1,178 patients: four studies included 789 children and three studies included a total of 389 adults.

Levodropropizine was compared with central antitussive in five studies [8–12] and against a placebo in two studies [13, 14].

### Data analysis

Due to the small number of clinical trials in the pediatric and adult population and the different clinical endpoints, the efficacy outcomes of the selected studies were standardized in order to compare the overall efficacy of levodropropizine versus control groups. Thus, the meta-analysis was performed after standardization of the overall efficacy variables assessed as endpoints in the eligible studies (i.e. reduction in cough frequency and severity, and number of night awakenings). For all the studies, original Absolute Mean Delta was calculated as the mean differences between baseline and final values of efficacy parameters in both groups, with the respective (approximate) standard deviations (SD) and the number of cases (N) studied in single treatment groups [15]. Standardized Mean Delta was calculated by means of the original Absolute Mean Delta (with their SD and N) and indicates a fraction or multiple of unitary standard deviations, expressed as standardized units [16, 17].

### Characteristics of included studies

The study of De Blasio et al. [10] was an observational one, carried out in 433 children (mean age 6 years) whose aim was to evaluate the efficacy of antitussive drugs in reducing the severity of acute cough associated with a URTI. A subgroup of 161 children received antitussive treatment with levodropropizine (N = 101) or central cough suppressants (codeine or cloperastine, N = 60).

In a double blind , two parallel groups, randomized study carried out by Kim et al. [11], the efficacy of levodropropizine was compared to the central antitussive dextromethorphan in 77 children (mean age 3 years) with acute or chronic bronchitis with non-recurrent or slightly recurrent cough treated for 2–3 days (oral t.i.d. administration). In a double blind, double-dummy, two parallel groups, randomized study Banderali et al. [8] evaluated the efficacy of levodropropizine compared to dropropizine, administered orally t.i.d. for 3 days, in the management of non-productive cough in 267 pediatric patients (2–14 years old).

The efficacy of levodropropizine vs. placebo in single oral dose for 4 weeks on nocturnal cough was investigated by Fiocchi et al. in a small double blind randomized study in 12 children with asthma [14]. In a double blind, two parallel groups , randomized study [12], Luporini et al. evaluated the efficacy of levodropropizine compared to dihydrocodeine (oral t.i.d. administration for 7 days) in the treatment of non-productive cough in 140 adults with primary lung cancer or metastatic cancer of the lungs. The double blind, double-dummy, parallel groups, randomized trial of Catena et al. [9], evaluated the therapeutic efficacy of levodropropizine compared to dextrometrorphan, administered orally t.i.d. for 5 days in 209 adults with moderate non-productive cough. The efficacy of levodropropizine vs. placebo (oral t.i.d administration for 3 days) on cough severity in 40 adult patients with bronchitis was evaluated in a double blind ,randomized , clinical trial carried out by Allegra et al. [13]. The main characteristics of published studies evaluating the antitussive efficacy of levodropropizine vs. control in children and adults are summarized in Tables 1 and 2, respectively.

**Table 1:** **Characteristics of clinical studies comparing levodropropizine to controls in children**

**Study**	**Design**	**Participants**	**Intervention vs. comparator**	**Condition**	**Outcomes**
De Blasio 2012	Observational study	Children N = 433 (161 valid for analysis) Mean age: 6,1 yrs	Levodropropizine vs. cloperastine/codeine	Acute cough associated with a URTI	Cough severity reduced by all antitussives
Kim 2002	RCT double-blind, two parallel groups Oral administration t.i.d. for 3 days	Children N = 77 (75 valid for analysis) Mean age: 3 yrs	Levodropropizine vs. dextromethorphan	Acute or chronic bronchitis with non-recurrent or slightly recurrent cough	Improvement in cough frequency and severity significantly higher with levodropropizine
Banderali 1995	RCT double-blind, double-dummy, prospective, two parallel groups, Oral administration t.i.d. for 3 days	Children N = 267 (258 valid for analysis) Age: 2–14 yrs	Levodropropizine vs. dropropizine	Non-productive cough	Significant decrease in cough frequency and night awakenings with both treatment
Fiocchi 1991	RCT double-blind, Oral administration in single dose for 4 weeks	Children N = 12 Age: 2–8 yrs	Levodropropizine vs. placebo	Asthmatic cough	Significant reduction in nocturnal awakening with levodropropizine

**Table 2:** **Characteristics of clinical studies comparing levodropropizine to controls in adults**

**Study**	**Design**	**Participants**	**Intervention vs. comparator**	**Condition**	**Outcomes**
Allegra 1988	RCT ,double-blind Oral administration t.i.d. for 3 days	Adults N = 40 Age: >13 yrs	Levodropropizine vs. placebo	Bronchitis cough	Higher reduction in cough severity with levodropropizine
Catena 1997	RCT , double-blind, double-dummy, two parallel groups Oral administration t.i.d. for 5 days	Adults N = 209 Age: 18–75 yrs	Levodropropizine vs. dextromethorphan	Moderate non-productive cough	Significant reduction in cough frequency with both treatments; Levodropropizine significantly more effective in reducing nocturnal awakenings
Luporini 1998	RCT, double-blind, two parallel gropus Oral administration t.i.d. for 7 days	Adults N = 140 > 18 yrs	Levodropropizine vs. dihydrocodeine	Lung cancer cough	Significant reduction in cough severity and nocturnal awakenings with both treatments

## Results

Table 3 shows the original Absolute Mean Delta (with SD and N) calculated for each efficacy parameter (cough frequency, cough severity, and night awakenings) assessed in each of the eligible clinical studies, both in levodropropizine and control groups in pediatric and adult patients.

**Table 3:** **Absolute and standardized mean delta** (**levodropropizine vs. controls**)

**Studies**, **parameters**	**Levodropropizine**			**Controls**			**Standardized Mean Delta**	**C.I. 95** **%**		**p**
	**Mean Delta**	**SD**	**N**	**Mean Delta**	**SD**	**N**		**Lower**	**Upper**	
Banderali, frequency	−8.4	17.3	130	−7.7	13.7	126	−0.045	−0.291	0.202	0.7216
Dong Soo, frequency	−1.3	1.14	38	−0.7	1.12	37	−0.525	−0.996	−0.055	0.0290
Banderali, nocturnal awakenings	−1.0	2.55	132	−1	2.46	126	0.000	−0.246	0.246	1.0000
Fiocchi, nocturnal awakenings	−1.06	0.81	12	−0.46	0.74	12	−0.747	−1.639	0.146	0.0966
DongSoo, severity	−1.2	1.0	38	−0.7	0.99	37	−0.495	−0.967	−0.028	0.0382
De Blasio, severity	−1.58	0.96	101	−1.1	1.13	60	−0.465	−0.792	−0.139	0.0055
Catena, frequency	−8.3	7.5	110	−8.2	7.75	99	−0.013	−0.287	0.260	0.9250
Catena, nocturnal awakenings	−2.7	1.8	80	−2.12	1.8	79	−0.321	−0.637	−0.005	0.0466
Luporini, nocturnal awakenings	−1	1.5	34	−1	1.4	29	0.0	−0.508	0.508	1.0000
Allegra, severity	−1.55	1	20	−0.8	1.11	20	−0.696	−1.362	−0.030	0.0410
Luporini, severity	−1.2	2.92	58	−1.2	3.2	65	0.0	−0.358	0.358	1.000

The results of the standardization of different efficacy variables, in order to make them comparable, are also shown in Table 3 as Standardized Mean Delta (with 95% C.I. and p between treatment groups).

**Figure 1:**
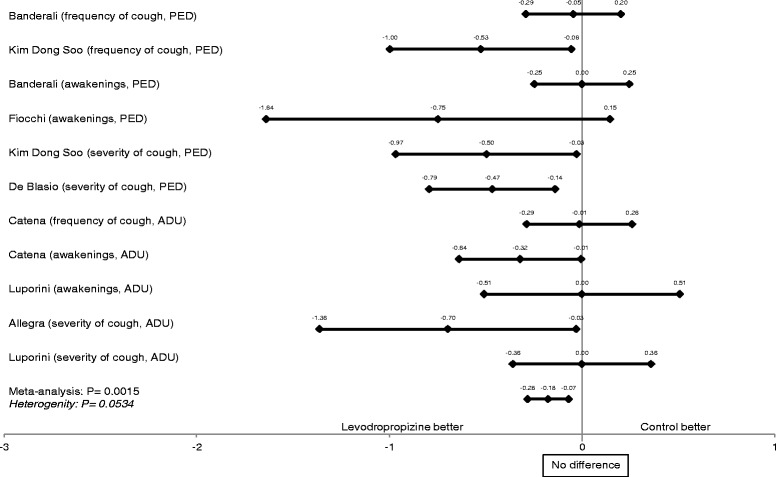
**Meta**-**analysis of the efficacy of levodropropizine vs. controls in pediatric and adult studies.**

The results of the meta-analysis of all standardized parameters, representing overall antitussive efficacy, showed a highly statistically significant difference in efficacy in favor of levodropropizine versus control treatments (including central cough suppressants), with p of 0.0015 (Table 4). The size of antitussive effect of levodropropizine vs. control treatments in the pediatric and adult setting is shown in the overall efficacy meta-analysis chart (Figure 1).

**Table 4:** **Meta**-**analysis of overall antitussive efficacy** (**levodropropizine vs. controls**)

**Levodropropizine versus Controls**	**Standardized Mean Delta**	**C.I. 95** **%**		**p**
		**Lower**	**Upper**	
	−**0.176**	−**0.282**	−**0.069**	**0.0015**

Concerning the estimated efficacy outcomes, levodropropizine was superior or equal to controls in all 7 clinical studies, also reaching a statistically significant difference (p < 0,05) in 4 studies.

In our meta-analysis, the test of heterogeneity for the efficacy outcome was not statistically significant (p = 0.0534).

## Discussion

Cough remains a serious unmet clinical problem [2]. It is a symptom of a range of diseases such as asthma, chronic obstructive pulmonary disease, GERD, or other conditions of unknown origin [2].

Managing the symptom of cough, regardless of whether the etiology is known, is also a challenge to even the most experienced health care provider [1].

The American College of Chest Physicians (ACCP) guidelines also recommend the use of peripheral cough suppressants such as levodropropizine in adult patients with cough due to acute or chronic bronchitis for the short-term symptomatic relief. These guidelines state that levodropropizine related to therapy of acute or chronic bronchitis has got the highest level of benefit, while the central antitussive drugs such as codeine and dextromethorphan show a lower level of benefit [18].

Recently, the European Medicines Agency (EMA) started a review of codeine-containing medicines when used for cough and cold in children. In fact, codeine is converted into morphine by CYP2D6 enzyme. It is well-known that some patients defined as ‘CYP2D6 ultra-rapid metabolizers’ convert codeine to morphine at a faster rate, resulting in higher levels of morphine in their blood leading to toxic effects such as breathing difficulties. EMA is still evaluating the available evidence on the benefit-risk balance of codeine-containing medicines when they are used for cough and cold in children [19]. FDA and MHRA recommended against the use of OTC products for cough and colds, as central antitussives in infants and young children and the American Academy of Paediatrics has advised against using dextromethorphan and codeine for treating cough in the pediatric population [20].

The major efficacy of levodropropizine in comparison to central antitussives has been recently demonstrated in a meta-analysis considering only children with cough of various origin (10).

This standardized meta-analysis of 7 published clinical studies, despite some limitations mainly linked to the small number of trials included in the analysis and the different efficacy variables assessed, provides an overview of the major comparative studies on levodropropizine in terms of efficacy both in the pediatric and adult setting, demonstrating a higher efficacy of levodropropizine.

## Conclusions

Levodropropizine is an effective antitussive drug both in children and adults, showing statistically significant better outcomes vs. central antitussive drugs in terms of overall efficacy in reducing cough intensity, frequency and night awakenings.

These positive results are particularly important considering that levodropropizine is a very well tolerated peripheral antitussive drug, while centrally-acting cough suppressants may be associated with serious side effects that limit their use, thus further reinforcing the favorable benefit/risk profile of levodropropizine in the management of cough.
